# Prevalence of hypertension and diabetes after exposure to extracorporeal shock-wave lithotripsy in patients with renal calculi: a retrospective non-randomized data analysis

**DOI:** 10.1007/s11255-018-1857-2

**Published:** 2018-05-21

**Authors:** Christian Daniel Fankhauser, Nilufar Mohebbi, Josias Grogg, Alexander Holenstein, Qing Zhong, Thomas Hermanns, Tullio Sulser, Johann Steurer, Poyet Cedric

**Affiliations:** 1 Department of Urology, University Hospital Zurich, University of Zurich, Zurich, Switzerland; 2 Division of Nephrology, University Hospital Zurich, University of Zurich, Zurich, Switzerland; 3 Department of Pathology of Molecular Pathology, University Hospital Zurich, University of Zurich, Zurich, Switzerland; 4 Cancer Data Science Group, Children’s Medical Research Institute, University of Sydney, Sydney, NSW Australia; 5 Horten Centre for Patient Oriented Research and Knowledge Transfer, University Hospital Zurich, University of Zurich, Zurich, Switzerland

**Keywords:** Kidney calculi, Lithotripsy, Treatment outcome, Adverse effects

## Abstract

**Purpose:**

To evaluate the association of shock-wave lithotripsy (SWL) for kidney stones and hypertension or diabetes.

**Methods:**

Patients with urolithiasis treated by SWL were retrospectively identified. To assess whether shock-wave application to the kidney is associated with long-term adverse effects, patients after SWL for kidney stones were selected as the main group of interest. Patients treated with shock waves for distal ureter stones only were chosen as a comparison group. A questionnaire was sent to all patients to assess the prevalence of hypertension and diabetes. The Swiss Health Survey (SHS) dataset was used as an additional comparison group.

**Results:**

After a median follow-up of 13.7 years, the odds ratio (OR) to report hypertension [OR 1.30 (95% CI 1.10–1.95)] or diabetes [OR 1.54 (95% CI 1.21–1.97)] was significantly higher in patients treated with SWL compared to the SHS dataset. In comparison with the kidney group, participants in the SHS had a significantly lower OR to report hypertension at follow-up [OR 0.79 (95% CI 0.65–0.95)], while the OR to report hypertension [1.16 (95% CI 0.79–1.70)] was not significantly different in the distal ureter group. For diabetes, a significantly lower [OR 0.60 (95% CI 0.46–0.78)] in the SHS group and a non-significantly lower [OR 0.68 (95% CI 0.38–1.22)] in the ureter group was noted compared to the kidney group.

**Conclusion:**

Compared to the SHS data set SWL was in general associated with hypertension and diabetes. However, no clear difference between patients after SWL to the kidney compared to SWL to the distal ureter was seen and thus the data do not support a causal relationship.

## Introduction

Since its introduction in the 1980s, extracorporeal shock-wave lithotripsy (SWL) quickly became the standard therapy for urinary calculi [1, 2]. Short-term side effects of SWL are well known and include renal hematoma, infectious complications, “steinstrasse” and renal colic caused by remaining calculi [3–5]. The question has been raised, whether SWL causes long-term damage to the kidney or adjacent organs (e.g., pancreas) [6, 7]. In our recent systematic review, we identified only weak evidence regarding potential long-term adverse effects like hypertension or diabetes after SWL [8]. Nevertheless, significant long-term effects may influence clinical decision making, in particular when ureterorenoscopy (URS) is available as an alternative first-line intervention for the treatment of kidney stones < 20 mm [9, 10]. In this study, we aimed to analyze whether SWL applied to the kidney is associated with a higher risk to develop hypertension or diabetes after long-term follow-up.

## Patients and methods

All patients with urinary calculi treated by SWL at our tertiary care center between 1993 and 2013 were retrospectively identified. Two different lithotripters were used during the study period: From the start of the study until 09/2007, the ESWL treatments were performed on a Dornier DL50 lithotripter (Dornier MedTech, Wessling, Germany). Subsequently, a Dornier DLS II (Dornier MedTech, Wessling, Germany) was in operation from 09/2007 until the end of this study.

**Fig. 1:**
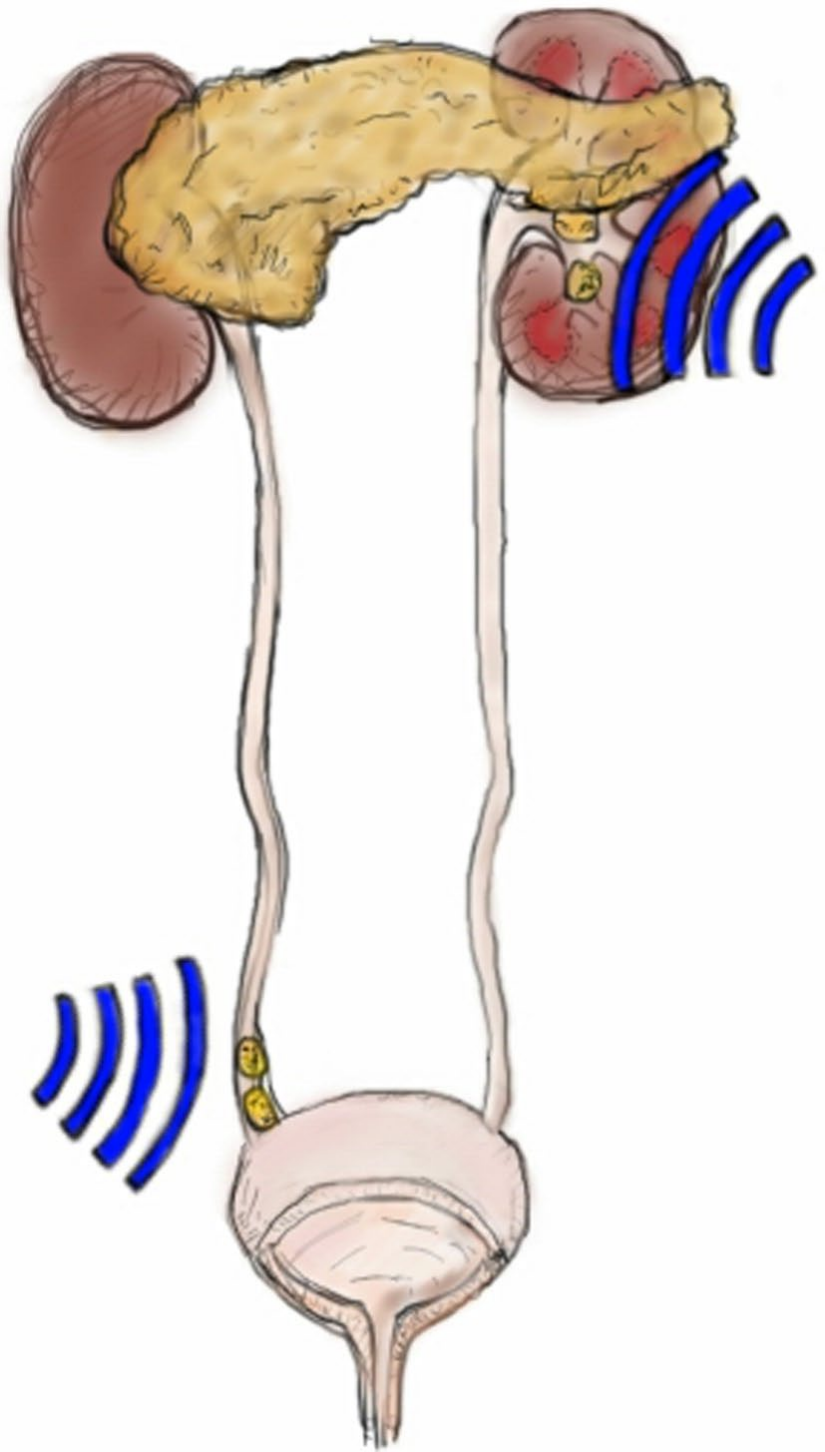
Anatomical illustration of the shock-wave-exposed organs. In the current study the proportion of long-term adverse effects in the kidney stone group was compared with the proportion in the distal ureter stone group

We hypothesized that the prevalence of hypertension and diabetes is increased in patients treated by SWL for kidney stones compared to patients treated by SWL for distal ureteral stones (Fig. 1), due to direct damage from shock waves to the kidney (hypertension) or the adjacent pancreas (diabetes). To assess whether SWL applied to the kidney is associated with long-term complications, patients were divided in two groups: The first group of patients consisted of patients treated by SWL for kidney stones (kidney group). Patients with distal ureter stones treated by SWL served as a second group (control group) as the kidneys were not exposed to shock waves. Patients with SWL treatments for upper or middle ureter stones were excluded as kidneys, and the pancreas might have been exposed to shock waves to some extent. Subsequently, patients treated by SWL for both kidney and distal ureter stones were excluded as well. After chart review, the following perioperative parameters for each patient were noted: Age, gender, number of SWL sessions and total of shock waves per session per patient.

Next, the study questionnaire was sent by mail to all included patients listed in our clinic records as being alive in 2015. The study questionnaire included the following questions: current weight, height, previous treatments for urolithiasis, current medication, diagnosis of or counseling for hypertension or diabetes. If patients did not respond after 3 weeks, the questionnaire was sent by mail one more time. As a comparison group, the study questionnaire data were compared with the Swiss Health Survey (SHS) data set [11]. The SHS selects individuals from the Swiss permanent population, meaning Swiss citizens and foreigners with a legal work permit aged 15 years and older, living in a private household. In 2012, 21.597 (53.1%) of 41.008 individuals participated in the survey. The survey included questions regarding the participants’ health state including hypertension and diabetes. Answers of the study questionnaire from patients with urinary calculi treated by SWL in the past (kidney and control group) were compared with each other, and additionally, the SHS data set served as a third comparison group.

Independent predictors of hypertension or diabetes at follow-up were identified by multivariable analysis using logistic regression using the following covariates: Age, gender, BMI, different patient/population groups (kidney group, distal ureter group and SHS) or number of shock waves to either the kidney or distal ureter. We first compared all patients who received an SWL treatment to the SHS data set. Second, we compared the three different groups to each other (model 1). Finally, we analyzed dose dependence between number of shock waves and the reported diagnosis of hypertension or diabetes at follow-up (model 2). The variables BMI and age were grouped into quartiles. Because of an interaction between age and gender for hypertension as outcome, the interaction term age (in quartiles)*gender was added to the model with hypertension as outcome.

The results for continuous normally distributed variables are expressed as means ± standard deviation (SD), and differences in patient characteristics between two groups were compared using Student’s unpaired *t* test. Continuous non-normally distributed variables are presented as median and interquartile ranges (IQR) and analyzed using the Mann–Whitney *U* test. The results for categorical variables are presented as percentage analyzed using Fisher’s exact test or Chi-square test, whenever appropriate. Odds ratios (ORs) with 95% confidence intervals (CI) were calculated. Statistical analysis was performed with IBM SPSS Statistics (Version 24.0, Armonk, New York, USA: IBM Corp.). All statistical tests were two-sided, and a p value of < 0.05 was considered significant.

## Results

**Fig. 2:**
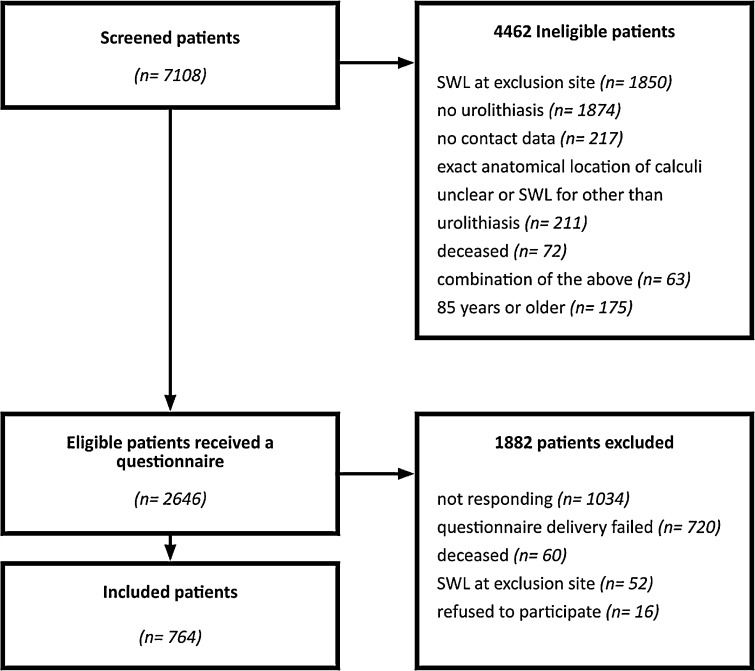
Participant flow diagram. Of 7108 patents who were selected for chart review and questionnaires were sent to 2646 patents fulfilling all inclusion and exclusion criteria. The final cohort included 764 patients

A total of 7108 patients were available for chart review. After review, 4335 (61%) patients did not meet the predefined inclusion criteria (Fig. 2): No SWL treatment for urolithiasis (1874), SWL treatment to the proximal/middle ureter or for kidney and distal ureter stones (1850), unclear anatomical location of the stone (221), age > 85 years (175), no contact data (90), death (72), several exclusion criteria (63). Eventually, questionnaires were sent to 2773 patients of which we had to exclude 856 patients because of: invalid contact data (720), death (60) or external retreatment for proximal/middle ureter or for kidney and distal ureter stones (52). For the final analysis 764/2646 (29%), patient questionnaires were available after a median follow-up of 13.7 years. A total of 585/764 (77%) patient questionnaires belonged to the SWL treatment group for kidney stones (kidney group), whereas 179/764 (23%) patient questionnaires belonged to the SWL treatment group for distal ureter stones.

The kidney group, the distal ureter group and the SHS data set differed regarding age, BMI and gender distribution (Table 1). The prevalence of hypertension and diabetes at follow-up for the kidney group, ureter group and SHS data set were 47.5, 49.4 and 27.5% and 14.1, 11.9 and 4.9%, respectively.

**Table 1:** Patient characteristics

	Kidney stone	Distal ureter	Swiss Health Survey
Number of patients	585	179	21 597
Age at follow-up (mean, years)	62.4 ± 14.4	63.8 ± 13.8	48.2 ± 18.6
Follow-up time (mean, years)	14.3 ± 6.1	15.7 ± 5.5	NA
BMI (mean, kg/m^2^)	26.3 ± 4.2	26.2 ± 4.1	24.6 ± 4.4
Gender (% female)	34.2	27.9	52.4
Number of SWL sessions (median)	1.0 [1, 2]	1.0 [1]	NA
SW applied (median)	3000 [3000–6000]	4000 [3000–4000]	NA
Arterial hypertension	47.5%	49.4%	27.5%
Diabetes mellitus	14.1%	11.9%	4.9%

*BMI* body mass index, *NA* not applicable, *SW* shock waves

A multivariable regression analysis adjusted for the significant confounders age, gender and BMI was performed to compare the SHS population to urolithiasis patients treated with shock-wave therapy. The odds to report hypertension (OR 1.30, 95% CI 1.10–1.95, *p* < 0.001) after a mean follow-up of 13.7 years was significantly higher in patients with at least one shock-wave treatment to any anatomical location. Furthermore, patients with at least one shock-wave treatment showed significantly higher odds (OR 1.54, 95% CI 1.21–1.97, *p* < 0.001) to report diabetes at follow-up.

To distinguish the effect regarding anatomical location another multivariable regression analysis adjusted for age, gender and BMI was performed. Compared to patients with shock waves to the kidneys (kidney group), participants in the SHS had a significantly lower OR to report hypertension at follow-up [OR 0.78 (95% CI 0.65–0.95), *p* = 0.014], while patients with shock waves to the distal ureter had a similar OR of 1.16 (95% CI 0.79–1.70), *p* = 0.458) (Table 2, model 1). Compared to the SWL kidney group, the OR to report diabetes at follow-up was 0.60 (95% CI 0.46–0.78), *p* < 0.001) in the SHS data set and 0.68 (95% CI 0.38–1.22), *p* = 0.199) in the distal ureter group (Table 3, model 1).

**Table 2:** Multivariable logistic regression analysis of prevalence of hypertension at follow-up

Model 1			Model 2		
Hypertension	OR (95% CI)	*p* value	Hypertension	OR (95% CI)	*p* value
Gender (male vs. female)	0.92 (0.78–1.10)	0.37	Gender (male vs. female)	0.92 (0.77–1.01)	0.371
Age		< 0.001	Age		< 0.001
1st quartile	Reference		1st quartile	Reference	
2nd quartile	2.08 (1.45–2.94)	< 0.001	2nd quartile	2.09 (1.48–2.95)	< 0.001
3rd quartile	4.47 (3.24–6.16)	< 0.001	3rd quartile	4.48 (3.25–6.17)	< 0.001
4th quartile	6.23 (4.51–8.56)	< 0.001	4th quartile	6.29 (4.56–8.67)	< 0.001
BMI		< 0.001	BMI		< 0.001
1st quartile	Reference		1st quartile	Reference	
2nd quartile	1.51 (1.36–1.69)	< 0.001	2nd quartile	1.51 (1.36–1.69)	< 0.001
3rd quartile	2.20 (2.00–2.44)	< 0.001	3rd quartile	2.20 (1.97–2.44)	< 0.001
4th quartile	4.33 (3.90–4.81)	< 0.001	4th quartile	4.33 (3.90–4.80)	< 0.001
Anatomical location		< 0.001			
SW to the kidney	Reference				
No SW	0.79 (0.65–0.95)	0.014			
SW to the distal ureter	1.16 (0.79–1.70)	0.458			
			Per 1000 SW to the kidney	1.05 (1.01–1.09)	0.016
			Per 1000 SW to the distal ureter	1.09 (1.00–1.18)	0.049

*BMI* body mass index, *SW* shock waves

**Table 3:** Multivariable logistic regression analysis of prevalence of diabetes at follow-up

Model 1			Model 2		
Diabetes	OR (95% CI)	*p* value	Diabetes	OR (95% CI)	*p* value
Gender (male vs. female)	1.81 (1.04–3.15)	0.035	Gender (male vs. female)	1.81 (1.04–3.15)	0.035
Age		< 0.001	Age		< 0.001
1st quartile	Reference		1st quartile	Reference	
2nd quartile	4.72 (1.62–13.74)	0.004	2nd quartile	4.75 (1.63–13.85)	0.004
3rd quartile	19.18 (7.14–51.53)	< 0.01	3rd quartile	19.27 (7.17–51.76)	< 0.001
4th quartile	44.26 (16.87–116.10)		4th quartile	45.50 (17.37–119.40)	< 0.001
BMI		< 0.001	BMI		< 0.001
1st quartile	Reference		1st quartile	Reference	
2nd quartile	1.27 (0.97–1.66)	0.78	2nd quartile	1.27 (0.97–1.66)	0.079
3rd quartile	1.68 (1.31–2.17)	< 0.001	3rd quartile	1.69 (1.32–2.18)	< 0.001
4th quartile	4.46 (3.54–5.62)		4th quartile	4.46 (3.54–5.62)	< 0.001
Anatomical location		< 0.001			
SW to the kidney	Reference				
No SW	0.60 (0.46–0.78)	< 0.001			
SW to the distal ureter	0.68 (0.38–1.22)	0.199			
			Per 1000 SW to the kidney	1.09 (1.04–1.14)	< 0.001
			Per 1000 SW to the distal ureter	1.00 (0.88–1.13)	0.961

*BMI* body mass index, *SW* shock waves

To test for a potential dose dependency for shock waves, a further multivariable regression analysis adjusted for age, gender and BMI to calculate the OR for increasing number of shock waves (per 1000 shock waves) was performed (model 2). Compared to the SHS data set, the odds to report hypertension was significantly and incrementally higher after every 1000 shock waves applied to the kidneys [OR 1.05 (95% CI 1.01–1.09), *p* = 0.016] or in every 1000 shock waves applied to the distal ureter [OR 1.09 (95% CI 1.00–1.18, *p* = 0.049] (Table 2, model 2). Similarly, the OR to report diabetes at follow-up was significantly higher in patients with more shock waves applied to the kidneys [OR 1.09 (95% CI 1.04–1.14), < 0.001] but not to the distal ureter [OR 1.00 (95% CI 0.88–1.13), *p* = 0.961] (Table 3, model 2).

## Discussion

This study shows two important results: First, patients with urolithiasis treated by shock waves at any location were at a higher risk to develop hypertension and diabetes during follow-up. Second, the odds to report hypertension or diabetes were not different in patients treated with shock waves for kidney stones compared to patients treated for distal ureter stones by SWL.

Regarding the first result, our multivariable regression analysis adjusted for known confounders confirmed that patients with urinary calculi are at a higher risk for long-term adverse effects including arterial hypertension [12–14] and diabetes mellitus [15]. Therefore, studies observing potential long-term adverse effects of stone treatment should ideally compare patients with urinary calculi with or without treatment and not patients treated with urinary calculi compared to the general population. Otherwise, two conditions including the underlying metabolic dysfunction as well as the SWL treatment will be responsible for the higher prevalence of hypertension or diabetes.

Regarding the second result, patients after SWL to the kidney showed similar odds to report hypertension or diabetes compared to patients after SWL to the distal ureter. Patients who had shock waves to the distal ureter were chosen as the main comparison group within SWL treated patients, as the distal ureter is as most far afield to the kidneys and pancreas and thus both organs were not exposed to shock waves in the distal ureter group. Taken together, our data do not support the hypothesis that SWL to the kidneys leads to a higher odds to report hypertension or diabetes after long-term follow-up. Our present findings are in line with our recent systematic review, in which the evidence for SWL causing hypertension or diabetes was weak [8].

Since our review in 2013, two further systematic reviews including the same studies [16, 17] and two cohort studies have been published. The first systematic review looked at new-onset diabetes after SWL for urinary stones and concluded that there is no association between SWL and new-onset diabetes [16]. The second review focused on long-term renal functions after SWL in the pediatric population and concluded that there is no evidence suggesting long-term damage [17]. The first cohort study by Pirola included 100 patients treated by SWL and found that creatinine values remained unchanged and the prevalence of diabetes and hypertension seems to be similar to the general population [18]. However, this study compared a SWL cohort of kidney stone patients with the general population. This limits its interpretation as patients with urolithiasis are in general at a higher risk to develop hypertension and diabetes during follow-up as discussed above. The second retrospective study by Denburg et al. [19] included 1319 patients treated by SWL with a follow-up of less than 4 years. They found that SWL was associated with a significant increased risk of hypertension. When they further stratified if SWL was applied to the kidney or ureter, only SWL to the kidney was significantly and independently associated with hypertension.

The result of our study and that of von Denburg have to be interpreted in the context of the study design. A major confounding variable may represent stone burden differences in the kidney and ureter group. In both studies, it was not possible to account for number and size of kidney stones. Therefore, patients with more and/or larger stones due to worse metabolic disease are more likely to be in the kidney stone group, which represent a possible confounder. We found that patients with an increasing number of shock waves (per 1000 shock waves) to the kidney were more likely to report hypertension or diabetes at follow-up. The observed dose dependence raises the question whether this association is causal. However, recurring treatment is more likely needed in patients with underlying metabolic dysfunction causing recurrent stone formation which again might be an important confounder.

Limitations of our study are the moderate questionnaire response rate and the unavailable information regarding hypertension or diabetes at time-point of SWL. Additionally, currently used lithotripter, newer settings (e.g., ramping [20, 21]) and co-current medications [22] have been discussed to reduce damage to the kidneys but were not used in this study. Nevertheless, in the absence of prospective data from larger cohorts with an appropriate follow-up, our study is a unique and valuable opportunity to study long-term adverse effects. Furthermore, our medical chart review minimizes the misclassification bias as no automatic code extraction was used.

## Conclusion

In conclusion, we recommend that physicians should not only counsel patients with urinary calculi regarding stone metaphylaxis but also regarding their increased risk to develop hypertension and diabetes. According to our data, SWL seems to be a safe procedure and there are no or only minimal long-term adverse effects like hypertension or diabetes.

## References

[1] Riedler I, Trummer H, Hebel P, Hubmer G (2003). Outcome and safety of extracorporeal shock wave lithotripsy as first-line therapy of lower pole nephrolithiasis. Urol Int.

[2] Zanetti GR, Montanari E, Guarneri A, Trinchieri A, Mandressi A, Ceresoli A (1992). Long-term followup after extracorporeal shock wave lithotripsy treatment of kidney stones in solitary kidneys. J Urol.

[3] Turk C, Petrik A, Sarica K, Seitz C, Skolarikos A, Straub M, Knoll T (2016). EAU guidelines on interventional treatment for urolithiasis. Eur Urol.

[4] Donaldson JF, Lardas M, Scrimgeour D, Stewart F, MacLennan S, Lam TB, McClinton S (2015). Systematic review and meta-analysis of the clinical effectiveness of shock wave lithotripsy, retrograde intrarenal surgery, and percutaneous nephrolithotomy for lower-pole renal stones. Eur Urol.

[5] Srisubat A, Potisat S, Lojanapiwat B, Setthawong V, Laopaiboon M (2014). Extracorporeal shock wave lithotripsy (ESWL) versus percutaneous nephrolithotomy (PCNL) or retrograde intrarenal surgery (RIRS) for kidney stones. Cochrane Database Syst Rev.

[6] Yu C, Longfei L, Long W, Feng Z, Jiping N, Mao L, Lin Q, Hequn C (2014). A systematic review and meta-analysis of new onset hypertension after extracorporeal shock wave lithotripsy. Int Urol Nephrol.

[7] Rezaee ME, Ward CE, Pollock M, Shetty SD (2017). Association between multiple chronic conditions and urolithiasis. Int Urol Nephrol.

[8] Fankhauser CD, Kranzbuhler B, Poyet C, Hermanns T, Sulser T, Steurer J (2015). Long-term adverse effects of extracorporeal shock-wave lithotripsy for nephrolithiasis and ureterolithiasis: a systematic review. Urology.

[9] Assimos D, Krambeck A, Miller NL, Monga M, Murad MH, Nelson CP, Pace KT, Pais VM, Pearle MS, Preminger GM, Razvi H, Shah O, Matlaga BR (2016). Surgical management of stones: American Urological Association/Endourological Society Guideline. Part II. J Urol.

[10] Fankhauser CD, Hermanns T, Lieger L, Diethelm O, Umbehr M, Luginbühl T, Sulser T, Müntener M, Poyet C (2018). Extracorporeal shock wave lithotripsy versus flexible ureterorenoscopy in the treatment of untreated renal calculi. Clin Kidney J.

[11] Volken T (2013). Second-stage non-response in the Swiss Health Survey: determinants and bias in outcomes. BMC Public Health.

[12] Alexander RT, Hemmelgarn BR, Wiebe N, Bello A, Samuel S, Klarenbach SW, Curhan GC, Tonelli M (2014). Kidney stones and cardiovascular events: a cohort study. Clin J Am Soc Nephrol.

[13] Madore F, Stampfer MJ, Willett WC, Speizer FE, Curhan GC (1998). Nephrolithiasis and risk of hypertension in women. Am J Kidney Dis.

[14] Madore F, Stampfer MJ, Rimm EB, Curhan GC (1998). Nephrolithiasis and risk of hypertension. Am J Hypertens.

[15] Obligado SH, Goldfarb DS (2008). The association of nephrolithiasis with hypertension and obesity: a review. Am J Hypertens.

[16] Deng T, Liao B, Tian Y, Luo D, Liu J, Jin T, Wang K (2015). New-onset diabetes mellitus after shock wave lithotripsy for urinary stone: a systematic review and meta-analysis. Urolithiasis.

[17] Akin Y, Yucel S (2014). Long-term effects of pediatric extracorporeal shockwave lithotripsy on renal function. Res Rep Urol.

[18] Pirola GM, Micali S, Sighinolfi MC, Martorana E, Territo A, Puliatti S, Bianchi G (2016). Evaluation of long-term side effects after shock-wave lithotripsy for renal calculi using a third generation electromagnetic lithotripter. Urolithiasis.

[19] Denburg MR, Jemielita TO, Tasian GE, Haynes K, Mucksavage P, Shults J, Copelovitch L (2016). Assessing the risk of incident hypertension and chronic kidney disease after exposure to shock wave lithotripsy and ureteroscopy. Kidney Int.

[20] Connors BA, Evan AP, Handa RK, Blomgren PM, Johnson CD, Liu Z, Lingeman JE (2016). Using 300 pretreatment shock waves in a voltage ramping protocol can significantly reduce tissue injury during extracorporeal shock wave lithotripsy. J Endourol/Endourol Soc.

[21] Skuginna V, Nguyen DP, Seiler R, Kiss B, Thalmann GN, Roth B (2016). Does stepwise voltage ramping protect the kidney from injury during extracorporeal shockwave lithotripsy? Results of a prospective randomized trial. Eur Urol.

[22] El-Nahas AR, Elsaadany MM, Taha DE, Elshal AM, El-Ghar MA, Ismail AM, Elsawy EA, Saleh HH, Wafa EW, Awadalla A, Barakat TS, Sheir KZ (2017). A randomised controlled trial evaluating renal protective effects of selenium with vitamins A, C, E, verapamil, and losartan against extracorporeal shockwave lithotripsy-induced renal injury. BJU Int.

