# Correction to: Prevalence of hypertension and diabetes after exposure to extracorporeal shock-wave lithotripsy in patients with renal calculi: a retrospective non-randomized data analysis

**DOI:** 10.1007/s11255-018-1932-8

**Published:** 2018-07-19

**Authors:** Christian Daniel Fankhauser, Nilufar Mohebbi, Josias Grogg, Alexander Holenstein, Qing Zhong, Thomas Hermanns, Tullio Sulser, Johann Steurer, Cédric Poyet

**Affiliations:** 1 Department of Urology, University Hospital Zurich, University of Zurich, Zurich, Switzerland; 2 Division of Nephrology, University Hospital Zurich, University of Zurich, Zurich, Switzerland; 3 Department of Pathology of Molecular Pathology, University Hospital Zurich, University of Zurich, Zurich, Switzerland; 4 Cancer Data Science Group, Children’s Medical Research Institute, University of Sydney, Sydney, NSW Australia; 5 Horten Centre for Patient Oriented Research and Knowledge Transfer, University Hospital Zurich, University of Zurich, Zurich, Switzerland

## Correction to: International Urology and Nephrology (2018) 50:1227–1233 10.1007/s11255-018-1857-2

The ninth author name was incorrectly published in the original publication. The correct name should read as ‘Cédric Poyet’.

